# Biomarker Supervised G-CSF (Filgrastim) Response in ALS Patients

**DOI:** 10.3389/fneur.2018.00971

**Published:** 2018-11-26

**Authors:** Siw Johannesen, Bettina Budeus, Sebastian Peters, Sabine Iberl, Anne-Louise Meyer, Tina Kammermaier, Eva Wirkert, Tim-Henrik Bruun, Verena C. Samara, Wilhelm Schulte-Mattler, Wolfgang Herr, Armin Schneider, Jochen Grassinger, Ulrich Bogdahn

**Affiliations:** ^1^Department of Neurology, University Hospital Regensburg, Regensburg, Germany; ^2^Lifedatascience Consulting, Schriesheim, Germany; ^3^Department of Hematology, Internal Medicine III, University Hospital Regensburg, Regensburg, Germany; ^4^Stanford Neuroscience Health Center, Palo Alto, CA, United States

**Keywords:** amyotrophic lateral sclerosis, granulocyte-colony stimulating factor, cytokines, hematopoietic stem and progenitor cells, HSPC, treatment

## Abstract

**Objective:** To evaluate safety, tolerability and feasibility of long-term treatment with Granulocyte-colony stimulating factor (G-CSF), a well-known hematopoietic stem cell factor, guided by assessment of mobilized bone marrow derived stem cells and cytokines in the serum of patients with amyotrophic lateral sclerosis (ALS) treated on a named patient basis.

**Methods:** 36 ALS patients were treated with subcutaneous injections of G-CSF on a named patient basis and in an outpatient setting. Drug was dosed by individual application schemes (mean 464 Mio IU/month, range 90-2160 Mio IU/month) over a median of 13.7 months (range from 2.7 to 73.8 months). Safety, tolerability, survival and change in ALSFRS-R were observed. Hematopoietic stem cells were monitored by flow cytometry analysis of circulating CD34^+^ and CD34^+^CD38^−^ cells, and peripheral cytokines were assessed by electrochemoluminescence throughout the intervention period. Analysis of immunological and hematological markers was conducted.

**Results:** Long term and individually adapted treatment with G-CSF was well tolerated and safe. G-CSF led to a significant mobilization of hematopoietic stem cells into the peripheral blood. Higher mobilization capacity was associated with prolonged survival. Initial levels of serum cytokines, such as MDC, TNF-beta, IL-7, IL-16, and Tie-2 were significantly associated with survival. Continued application of G-CSF led to persistent alterations in serum cytokines and ongoing measurements revealed the multifaceted effects of G-CSF.

**Conclusions:** G-CSF treatment is feasible and safe for ALS patients. It may exert its beneficial effects through neuroprotective and -regenerative activities, mobilization of hematopoietic stem cells and regulation of pro- and anti-inflammatory cytokines as well as angiogenic factors. These cytokines may serve as prognostic markers when measured at the time of diagnosis. Hematopoietic stem cell numbers and cytokine levels are altered by ongoing G-CSF application and may potentially serve as treatment biomarkers for early monitoring of G-CSF treatment efficacy in ALS in future clinical trials.

## Introduction

Amyotrophic lateral sclerosis (ALS) is a life threatening neurodegenerative disorder characterized by premature loss of upper and lower motoneurons in the adult brain and spinal cord (1). The life time risk of ALS is below one in 400 individuals (2), the incidence is 2–3 per 100,000 in Europe (3). The unmet medical need in ALS patients is underlined by a median survival of 29.8 months from symptom onset, and of 15.8 months from diagnosis (4). Only modest treatment effects have been observed by riluzole (5) and edaravone (6).

In view of the great heterogeneity of disease etiology, neuronal damage likely results from many different pathologic changes, including neuroinflammation (3). Neurodegenerative processes with altered homeostasis, protein accumulation and cell death generates neuroinflammation, and central nervous system (CNS)-resident immune cells such as astrocytes and microglia trigger neuroinflammation and neurodegeneration (7). Inflammation may arise reactive to ALS-related CNS alterations, but also play an initial role and trigger both onset of disease and further accelerate progression of ALS (3). A complex, cytokine-mediated crosstalk between CNS and systemic immune cells regulates immune responses to either pro- or anti-inflammatory states, which evolve over time (7).

Granulocyte-colony stimulating factor (G-CSF) is a 20-kDa glycoprotein and a well characterized growth factor that plays a key role in production, mobilization, and differentiation of hematopoietic stem cells (8, 9). It is a widely used compound for treatment of neutropenia and for mobilization of CD34^+^ hematopoietic stem cells prior to bone marrow transplantation. G-CSF enhances immunocompetence and has systemic anti-inflammatory effects (10). G-CSF is safe and well tolerated; most common side effects are moderate bone pain and musculoskeletal pain in 20–30% of patients, rarely splenomegaly and allergic reactions (11). Aside from hematopoietic functions, G-CSF acts as a neuronal growth factor in the CNS and possesses neuroprotective and -regenerative properties (12, 13). G-CSF passes the intact blood brain barrier, and its receptor is widely expressed within the CNS (12). G-CSF is thought to be neuroprotective through anti-apoptotic effects (12, 14), it induces neural differentiation, supports neurogenesis, contributes to re-endothelialization and arteriogenesis (12, 15). Systemic G-CSF induced hematopoietic stem cells may contribute on a direct cellular level in neurodegeneration by migration to the CNS (16, 17), where they may offer trophic support and modulate the local CNS immune system (17, 18). Observing G-CSF induced systemic hematopoietic stem cells may also shed light upon direct G-CSF effects on neural cells and stem cells as a surrogate system. Furthermore, G-CSF modulates monocyte function and attenuates the neuroinflammatory cascade (13). An interesting bone marrow-brain connection has been shown as G-CSF induced bone marrow derived cells migrate to CNS and express microglial phenotype in a mouse model of cranial irradiation. This was associated with a better functional outcome and suggested to facilitate neuroprotection by direct effects on resident CNS cells as well as modulation of cellular microenvironment in neurovascular niches (15). Angiogenic factors may promote neurogenesis through direct effects on neuronal cells (19) and indirectly by angiogenic support of the highly vascularized neurogenic zones. G-CSF improved motor function and survival in mouse models of ALS (20–22). Small trials with G-CSF treatment in ALS patients demonstrated excellent tolerability and safety (23–25), with modulation of immune parameters (26), and possible minor benefits detected by neuroimaging (27). In summary, G-CSF exerts multiple physiological effects within the CNS and may be a potent modulator of different functions relevant to ALS pathophysiology (13). Importantly, from *in vitro*, mouse model and human exploratory evidence the mode of action most relevant for potential treatment effects cannot with certainty be concluded.

Due to the paucity of available treatment options we provided individual, off-label G-CSF treatment to ALS patients. G-CSF, considering its multimodal systemic and CNS effects, may be a promising treatment option in view of the etiopathological and clinical heterogeneity of ALS. Biomarkers are measurable indicators of disease and/or intervention and may be useful in monitoring long-term degenerative or reparative processes within the CNS. In view of the above-discussed complexity of ALS, it seems unlikely that a single biomarker can sufficiently reflect treatment effects on disease progression. We therefore used a panel of pro- and anti-inflammatory blood parameters, angiogenic factors, as well as hematopoietic stem cell markers. Monitoring pro-differentiation and -mobilization effects on hematopoietic stem cells may serve as a proxy for G-CSF activity on neural stem cells in individual patients and/or reflect direct and indirect beneficial effects of mobilized hematopoietic stem cells. Observing a panel of peripheral cytokines may reveal system wide immune and inflammatory status relevant for peripheral-CNS crosstalk.

G-CSF is known to be a safe stem-cell mobilizing agent. We investigated whether the number of mobilized hematopoietic stem cells is different in G-CSF treated ALS patients of longer versus shorter survival. Secondly, we were interested in whether baseline cytokine levels are associated with survival of G-CSF treated ALS patients. Lastly, we sought to explore hematopoietic stem cells and cytokine level alterations during G-CSF treatment.

## Methods

### Patients, procedures and ethics

Treatment with G-CSF was offered to 36 patients seen at the University of Regensburg with definite or probable ALS according to the revised El Escorial criteria (28). As this was not a prospective clinical trial, the use of formal exclusion criteria was not considered appropriate. However, neither patients with a current or past history of neurologic disease other than ALS, nor patients participating in any interventional study were offered this treatment option. Individual treatment of ALS patients and retrospective evaluation was done after written informed consent. The ethics committee of the University of Regensburg approved a retrospective analysis (ethics approval: 15-101-0106 and 14-101-0011). The principles of the Declaration of Helsinki (World Medical Association, revised version 2013) were strictly adhered to. Survival was defined as the time between diagnosis and death from confirmed ALS-related complications, including suicide.

**Figure 1:**
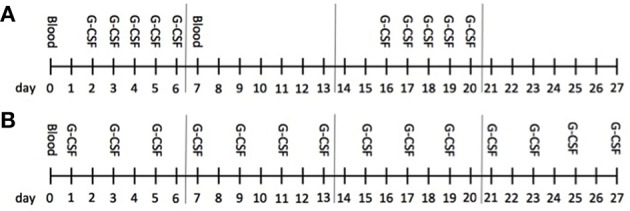
Treatment course. The monthly protocol is illustrated; this schedule was repeated and individually adapted over the long-term treatment. G-CSF was administered subcutaneously. Mainly, patients received G-CSF either as a 5-day bolus **(A)** once (1st week) or twice (1st and 3rd week) or continuously **(B)** on single days up to every second day; G-CSF was administered in one or two doses á day. Blood was obtained before treatment onset at baseline (d0) and then once á month (d0) in patients receiving G-CSF continuously, and before (d0) as well as after a 5-day treatment (d7) in patients on bolus application. Cytokines were analyzed at d0 in both groups and at d7 in bolus treatment in the first month (baseline), then at the 3rd and 6th month during ongoing treatment.

ALS patients were treated with subcutaneous injections of recombinant human G-CSF (Filgrastim) on an outpatient basis. Dose and application modes were adapted individually upon initiation and over time (Figure 1; Table S1). Adaption was made with the intent to maximize patient wellbeing and safety in the presence of any emerging safety signals, and with the aim of increasing efficacy as monitored by levels of mobilized hematopoietic stem cells, a potential individual marker of biological activity of G-CSF. This resulted in heterogeneous treatment schemes. The intervention and evaluation was initiated in January 2010 and is still ongoing. The data were analyzed up to March 2017. The treatment was provided by the hospital and not funded by a pharmacological company or other external source. No external or internal funding sources were involved in patient selection, study design, data analysis or interpretation.

Patient safety was analyzed at baseline (initiation of treatment) followed by monthly control visits with clinical examinations, blood counts, cytokines, blood smears and estimation of bone marrow function. We conducted baseline spleen sonography with follow-ups upon dose escalation. Clinical ALS progression was monitored using the established ALSFRS-R (29). If patients were not able to continue visits and treatment, patient survival was monitored by phone calls to patients, their families and general practitioners.

Changes in pro- and anti-inflammatory immune profiles were evaluated at baseline, at 3 months, and then every 6 months throughout treatment by multiplex electrochemoluminescence with the panel assay V-PLEX Human Biomarker 40-Plex Kit (MesoScale Discovery®, Maryland, USA). This industry standard panel has been validated in different immune related and non-immune diseases (manufacture's information). In patients receiving G-CSF on five consecutive days, evaluations of cytokine levels in the peripheral blood were conducted twice a month, before (day 0) and after G-CSF application (day 7). In patients receiving G-CSF twice a week or every second day, analyses were conducted on a monthly basis 1 day after application. Peripheral blood serum was collected during regular visits at the hospital and immediately stored at −20°C for cytokine assays. For each assay, 25 μl of serum samples were used and test carried out in duplicates, according to the manufacturer's instructions.

We analyzed white blood cells including cell differentiation, platelet and red blood cell counts, and hemoglobin levels with an automatic cell counter (Sysmex®, Kobe, Japan). Peripheral blood smears were done on a 3-month basis by light microscopy. Peripheral blood CD34^+^ and CD34^+^CD38^−^ hematopoietic stem and progenitor cells (HSPC) were analyzed by flow cytometry as earlier described by our group (25). In short, 1 ml donor blood was lysed in 9 ml NH_4_Cl lysis buffer and cells were then stained for 30 min at 4°C with combinations of anti-CD45-FITC (clone HI30, BD Pharmingen, Franklin Lakes, NJ, USA), CD34-APC (clone 581, Biolegend, San Diego, CA, USA) and CD38-PE (clone HIT2, BioLegend) monoclonal antibodies. Analysis was performed on a Becton Dickinson CALIBUR flow cytometer (BD, East Rutherford, NJ, US).

### Calculations and statistics

Findings of immune parameters from three time points, baseline (initiation of treatment), 3 months and 6 months were selected for analysis. As patients did not always visit the outpatient clinic on the exact days of the given time points, the time points had to be defined as time periods. When assessing the ALSFRS-R at baseline, data from day of treatment initiation ±28 days were included. For baseline measures of blood counts, stem cell mobilization parameters and cytokines, only data obtained before the first G-CSF application were selected. The 3-month time point was defined as day 45–134 and the 6-month time point as ranging from day 135 to 224. If patients visited more than once during these time periods, the day closest to the intended time point was selected.

The immediate effects of G-CSF treatment on peripheral levels of cytokines, hematopoietic stem cells and blood counts were assessed by comparing respective levels 2 days before and 1 day after a 5-day treatment course with G-CSF. We then explored different patterns of immune responses depending on individual survival. Survival time was defined as time elapsed from day of diagnosis to day of death or day of last observation in the case of censoring. For this purpose, G-CSF treated patients were divided into two groups based on their survival being longer or shorter than 30 months from diagnosis, as this was a time point that separated the patients into two equal-sized groups. At the point of database closure, patients who were still alive were censored and included in the “long survival” group if they had been observed for over 30 months (*n* = 7). Patients who were alive and had not yet been observed for over 30 months were not considered for this analysis (*n* = 3). The same censoring was applied for correlation analysis. We then retrospectively analyzed baseline levels of cytokines, hematopoietic stem cells and blood counts in the long and short survivor groups and further correlated survival with cytokines upon treatment initiation.

R or GraphPad Prism 7 was employed for statistical analysis and graph design. Correlations were analyzed using two-tailed Pearson correlation and presented with correlation coefficient (r), coefficient of determination (R^2^) and *p*-value. Comparisons were made with Mann-Whitney test and paired Wilcoxon test. Data were considered significant at *p* ≤ 0.05. A trend was noted at *p* ≤ 0.1. Comparisons were corrected for multiple testing by false discovery rate approach (FDR, two-stage step-up method of Benjamin, Krieger and Yekutieli with desired FDR (Q) at 10%) and considered a discovery at FDR-adjusted *p*-value (q) < 0.1. We used an Area Under the Curve (AUC) approach to estimate mobilization of hematopoietic stem cells after G-CSF treatment over time. Stem cell measurements before and after G-CSF dosing were available for patients on the 5-day treatment scheme. For better comparability regarding long and short survival times after diagnosis, we selected patients with ongoing 5-day treatment over the first 4 months. All patient measurements were used in the calculation. If patients had fewer data points, their mean AUC value calculated from all data points was applied (in the case of one patient). The AUC value was calculated with the *auc* function of the R-package “flux” (Jurasinski, Koebsch, Guenther and Beetz, 2014). The baseline value at day 0 or from any day prior to treatment start was used as threshold for the calculation.

## Results

### Demographics, intervention and safety

36 caucasian ALS patients (25 male, 11 female, 28 limb onset, 8 bulbar onset, mean age 51.9 years, mean ALSFRS-R on initiation 38/48) were treated with G-CSF in addition to riluzole treatment. We here report on individual treatment on a named patient basis—consequently, treatment schemes were heterogeneous. Dose and application modes were adapted individually upon initiation and over time (Table S1). In summary, G-CSF was injected subcutaneously in a dose-range from 90 to 2160 Mio IU per month (900–21,600 μg/month), with a mean dose of 464 Mio IU/month (4,640 μg/month). Application modes ranged from once weekly to every second day in an ongoing individually tailored manner. The median duration of treatment was 13.7 months (mean 16.7 months; range from 2.7 to 73.8 months) (Table 1, Figure 1).

**Table 1:** Demographics and intervention in G-CSF treated ALS patients.

**ALS patient**	**Age (years)**	**Gender**	**ALSFRS-R baseline**	**Site of onset**	**Time diagnosis to treatment (days)**	**Dose G-CSF (mean; range (MioIE/month))**	**Treatment duration (months)**	**Survival (months) from diagnosis**
1	50	F	38	Limb	498	150 (150–150)	19	36.2
2	42	M	32	Limb	619	280 (150–300)	31	52.2
3	77	M	21	Limb	759	173 (150–240)	5	33.4
4	68	F	39	Bulbar	29	150 (150–150)	3	3.9
5	67	M	33	Limb	439	260 (150–300)	20	56.6
6	26	M	33	Limb	486	485 (150–1170)	74	89.7*
7	50	F	33	Limb	536	240 (240–240)	7	25.4
8	73	M	41	Limb	270	166 (150–240)	11	21.4
9	50	M	28	Limb	393	133 (90–150)	7	21.4
10	56	M	37	Limb	770	242 (150–300)	14	40.0
11	41	M	38	Limb	24	287 (150–300)	27	36.3
12	35	F	40	Bulbar	115	296 (240–300)	14	63.7*
13	48	F	46	Limb	38	216 (150–300)	14	29.7
14	43	M	44	Limb	61	561 (192–768)	45	47.3
15	65	F	32	Limb	81	192 (192–192)	14	18.3
16	51	F	42	Limb	101	225 (150–300)	3	6.4
17	60	F	38	Limb	21	192 (192–192)	9	11.5
18	58	M	44	Limb	45	311 (240–480)	25	25.4
19	46	M	46	Limb	249	150 (150–150)	26	34.7
20	50	M	-	Limb	1	198 (192–240)	8	8.0
21	27	M	44	Limb	53	301 (150–600)	39	71.3*
22	45	M	48	Limb	26	666 (240–1296)	37	39.1*
23	55	M	40	Bulbar	26	263 (150–300)	3	41.9*
24	61	M	44	Limb	66	375 (150–510)	5	9.2
25	60	M	40	Bulbar	135	602 (240–816)	19	23.0
26	65	F	30	Bulbar	122	563 (240–900)	7	11.3
27	43	F	41	Limb	338	628 (240–720)	14	35.7*
28	60	M	42	Limb	23	589 (480–720)	11	12.1
29	45	F	28	Limb	493	535 (150–720)	6	29.6
30	47	M	29	Limb	396	585 (450–720)	5	19.3
31	50	M	40	Limb	23	667 (240–720)	8	13.7
32	39	M	41	Bulbar	343	1015 (450–1170)	20	31.7*
33	56	M	42	Bulbar	525	744 (450–1056)	11	35.6
34	59	M	38	Bulbar	52	1044 (450–1440)	14	#
35	69	M	39	Limb	62	1344 (450–2160)	16	#
36	35	M	38	Limb	288	1141 (300–1440)	14	#
Mean (SD)	51.9 (12.2)	11 F 25 M	38/48 (6.1)	28 Limb 8 Bulbar	236.3 (231.4)	222.7 (104.1)	16.7 (14.4)	25.5 (14.3) in *deceased* patients

Patients marked by # or ^*^ were alive upon closure of data admission. Patients who had been observed for less than 30 months at time of closure of data admission are marked by #. The sign ^*^ indicates patients, who at time of closure of data admission, had been observed more than 30 months from diagnosis. Baseline ALSFRS-R was not available in one patient, marked by -.

Long-term outpatient treatment with G-CSF was generally well tolerated in ALS patients and compliance was excellent. Minor adverse events were mild to moderate bone pain after G-CSF injection and leukocytosis. One patient experienced an episode with heat sensation, lightheadedness, and 15 min. of dyspnea on 1 day of drug application 39 months into G-CSF treatment. Due to the possibility of drug-related intolerance or mild allergic reaction, G-CSF was discontinued in this patient; antibodies against G-CSF were not detectable. This patient was switched from Filgrastim to Pegfilgrastim, a PEGylated form of recombinant human G-CSF, from his 46th to 53rd month after initiation, and then ended the off-label treatment without further adverse reactions. As expected, mild to moderate splenomegaly evolved during ongoing G-CSF treatment in most patients. Without any further symptoms or complications, the mean spleen width increased from 4.3 to 4.9 cm and length from 10.7 to 12.1 cm during treatment. There were no severe adverse events (SAE), and no signs for pre-malignant transformation in peripheral blood smears.

Baseline hematology showed no abnormalities in our patients. G-CSF mobilizes neutrophil leukocytes as well as CD34^+^ and CD34^+^CD38^−^ hematopoietic stem and progenitor cells (HSPC) from the bone marrow into the peripheral blood. Leukocyte counts increased significantly in all treated patients, from an initial mean of 6.9 × 10^3^/μl to 48.2 × 10^3^/μl (range 8.3–118.7 × 10^3^/μl, *p* < 0.0001) after G-CSF application. A predicted increase in the average percentage of neutrophils (from 64.8 to 87.3%, *p* < 0.0001) was accompanied by a relative decrease in lymphocytes (from 24.1 to 7.0%, *p* < 0.0001), monocytes (from 8.8 to 4.7%, *p* < 0.0001) and eosinophils (from 1.8 to 0.7%, *p* < 0.0001) as well as a small decrease in red blood cell count (from 5.03 to 4.83 × 10^3^/μl, *p* < 0.0001), hemoglobin level (from 14.9 to 14.4 g/dl, *p* < 0.0001) and hematocrit (from 44.2 to 43.6, *p* = 0.0362) during monitoring (all comparisons by paired *t*-test, two-tailed *p*-value. Figure S1). There were no significant changes in basophiles and platelet count during monitoring. The fold increase of CD34^+^ and CD34^+^CD38^−^ HSP cells in peripheral blood served as an indicator of mobilization efficiency and was determined by comparing cells at baseline to cells after mobilization. The mobilization efficacy was heterogeneous with high intra- and inter-personal variability (data not shown).

### G-CSF-mediated stem cell mobilization was associated with survival of ALS patients

**Figure 2:**
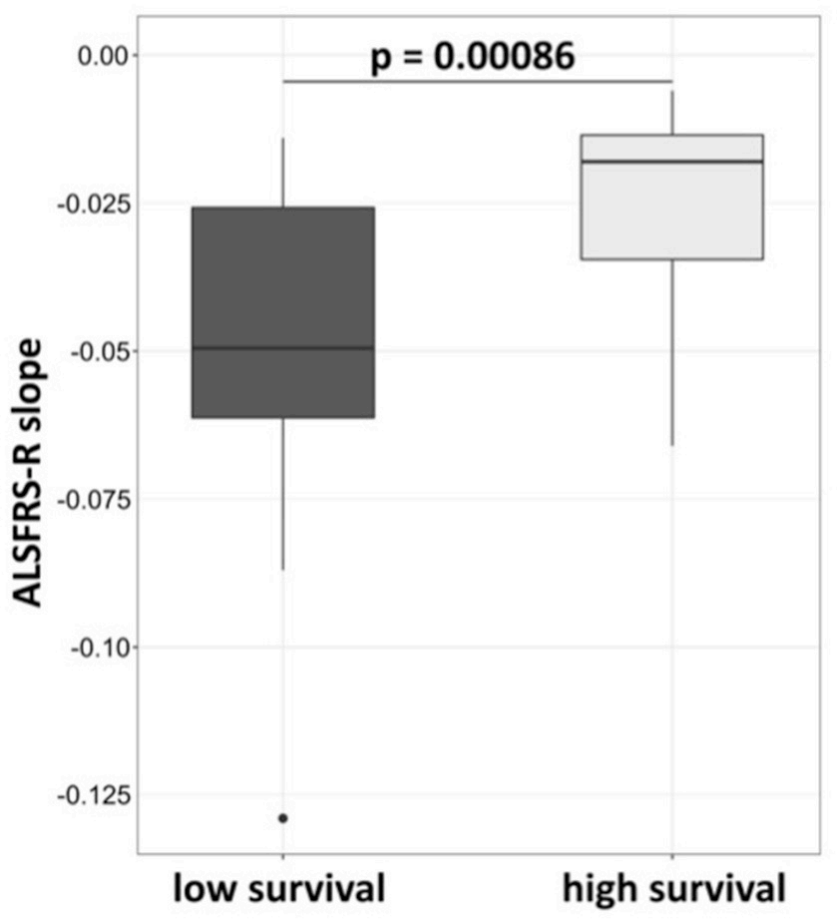
ALSFRS-R decline is less rapid in patients who survive longer than 30 months. Patients were assigned to high survival group at survival longer or at 30 months, and to low survival group at survival below 30 months from diagnosis. The slope was calculated by robust calculation of the ALSFRS-R measurements. Median slope in the high survival group was −0.019 and −0.05 in the low survival group. Wilcoxon test, *p*-value 0.00086.

Twenty-six of thirty-six G-CSF treated patients deceased between January 2010 and March 2017. 10 patients were alive, of which 6 were still treated with G-CSF. The patient who had suffered from a possible allergic reaction was regularly seen at the clinic. Three patients ended G-CSF treatment at days 82, 420 and 427, and were all lost to follow up. The overall median survival of *deceased* patients was 24.2 months from diagnosis (mean 25.5; range 3.9–56.6 months). For further analysis, patients were divided into two equally sized groups by survival being longer or shorter than 30 months from diagnosis. Patients, who were alive at the time of database closure, were considered for this analysis had they been observed for at least 30 months. The mean (median) survival differed in the two survival groups: 46.59 (39.55) months, SD 16.34 and 17.04 (18.30) months, SD 8.16 (two-tailed *p*-value < 0.0001; Mann-Whitney *t*-test). The ALSFRS-R slope over time was significantly flatter in longer surviving patients (Wilcoxon test, *p* = 0.00086; Figure 2). Long survivors were younger (mean age 46.8 vs. 56.5 y, unpaired *t*-test, *p* = 0.0163) and had a longer latency between diagnosis and treatment onset (mean 333 vs. 163 days, unpaired *t*-test, *p* = 0.0377). Their clinical function upon treatment initiation was not significantly different (mean ALSFRS-R 38.6/48 vs. 37.3/48). Further, longer surviving patients were less frequently female (18.8 vs. 47.1%), but had similar occurrence of bulbar onset of disease (18.8 vs. 17.6%) (Table 1).

**Figure 3:**
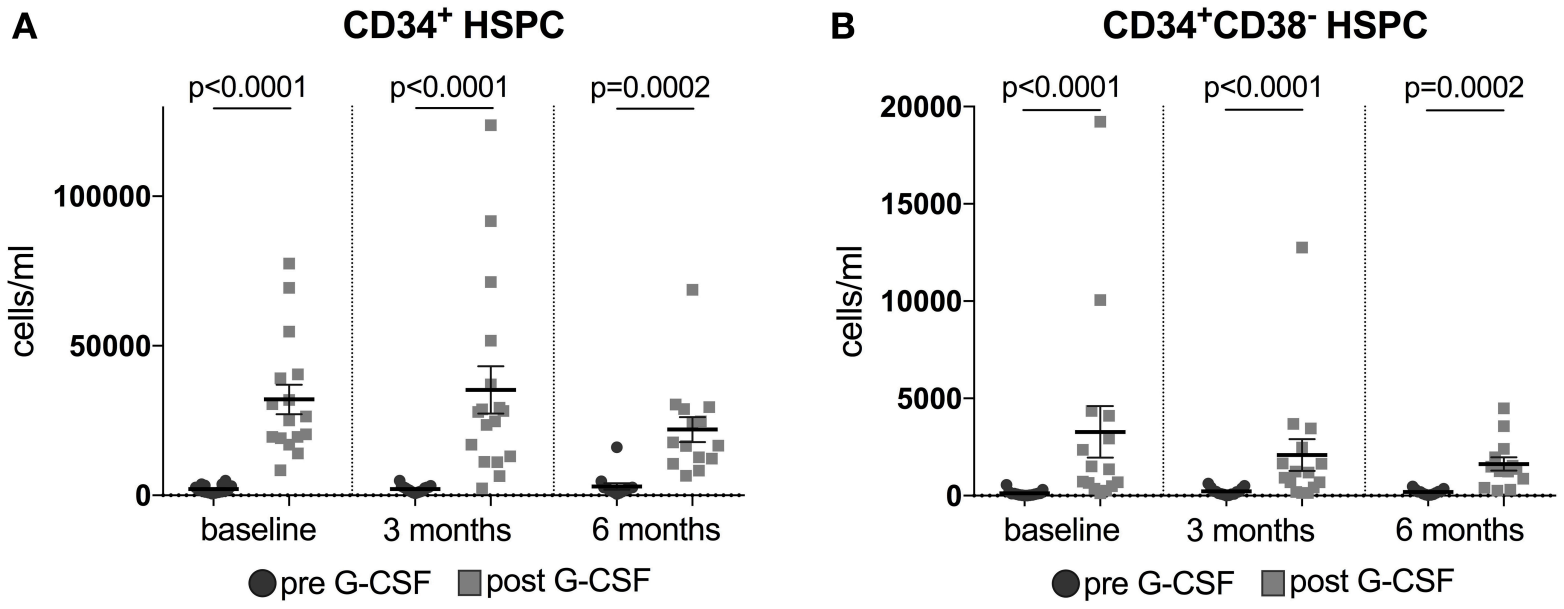
**(A,B)** Mobilization of hematopoietic stem cells (HSPC) in G-CSF treated ALS patients. Plotted are CD34^+^ (Figure 2A) and CD34^+^CD38^−^ HSPC (Figure 2B) in blood 2 days before (d0) and 1 day after (d7) daily application of G-CSF over 5 days in 16 (for CD34^+^)/15 (for CD34^+^CD38^−^) patients at baseline, in 17 patients after 3 months, and in 14 (for CD34^+^)/13 (for CD34^+^CD38^−^) patients after 6 months of treatment. Data are presented as scatter dot plot with mean + SEM. Paired Wilcoxon *t*-test, significance was taken at *p* < 0.05 (two-tailed). *T*-tests were corrected for multiple testing by FDR-adjusted *p*-values (*q*-values), discovery is indicated by *q* < 0.1. In CD34^+^ and CD34^+^CD38^−^ HSPC at all time points: *q*-value = 0.0002.

**Figure 4:**
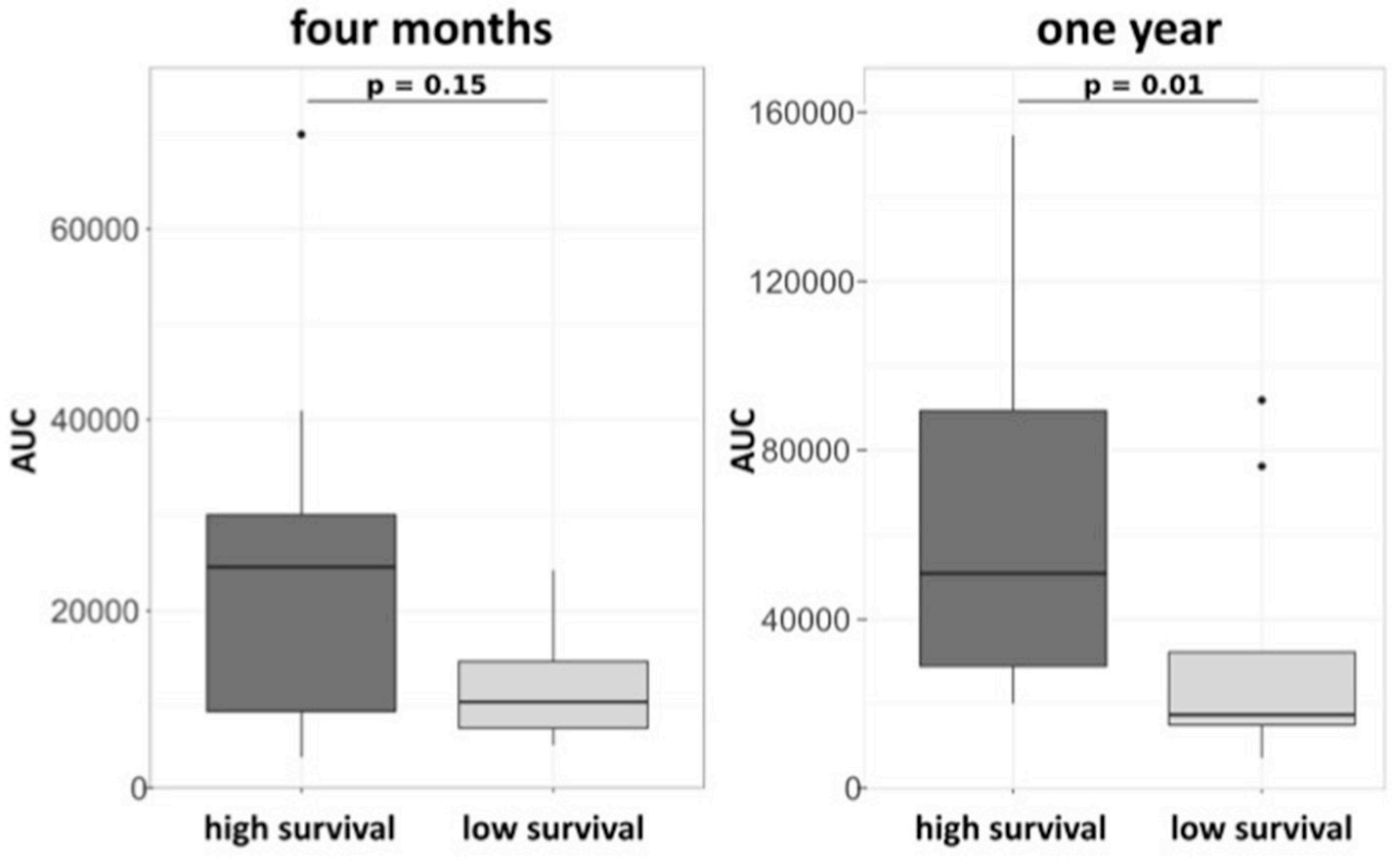
Mobilization of CD34^+^CD38^−^ HSPC is associated with survival in ALS patients on a 5-day treatment scheme with G-CSF. Area under the curve (AUC) approach on blood HSPC over 4 months **(A)** and 1 year **(B)** in patients treated with 5-day application of G-CSF (*n* = 20). Patients were assigned survival groups dependent on survival being longer (high survival *n* = 8) or shorter (low survival) than 30 months from diagnosis.

G-CSF is known to mobilize HSPC into the peripheral circulation. CD34^+^ and CD34^+^CD38^−^ HSPC were evaluated in the sera of patients 2 days before (day 0) and 1 day after (day 7) a 5-day treatment course with G-CSF at baseline, 3 months and 6 months. G-CSF led to a sustained increase of CD34^+^ and CD34^+^CD38^−^ HSPC at all time points (Figure 3). In patients treated with ongoing 5-day courses of G-CSF *t*-tests displayed no significant reductions in mobilization of CD34^+^ and CD34^+^CD38^−^ HSPC when comparing the respective levels after G-CSF treatment at baseline and after 3 and 6 months of treatment (mean number of CD34^+^/ml at baseline 30307, at 3 months 35250, at 6 months 22017; mean number of CD34^+^CD38^−^/ml at baseline 3092, at 3 months 2089, at 6 months 1632, all Wilcoxon paired *t*-test, all *p*-values not significant; Figure S2). However, we found a different capacity to mobilize hematopoietic stem cells in patients surviving longer or shorter than 30 months from diagnosis. This was analyzed by Area Under the Curve (AUC) approach to mobilized CD34^+^CD38^−^ cells within the first year of G-CSF treatment in 19 available patients, who all received ongoing 5-day treatment. Longer surviving patients displayed a significantly superior mobilization of CD34^+^CD38^−^ cells under G-CSF application at 1 year of treatment. At 4 months this difference was borderline significant (trend) (Figure 4).

### Short and long-term survivors differed in their baseline cytokine levels

**Figure 5:**
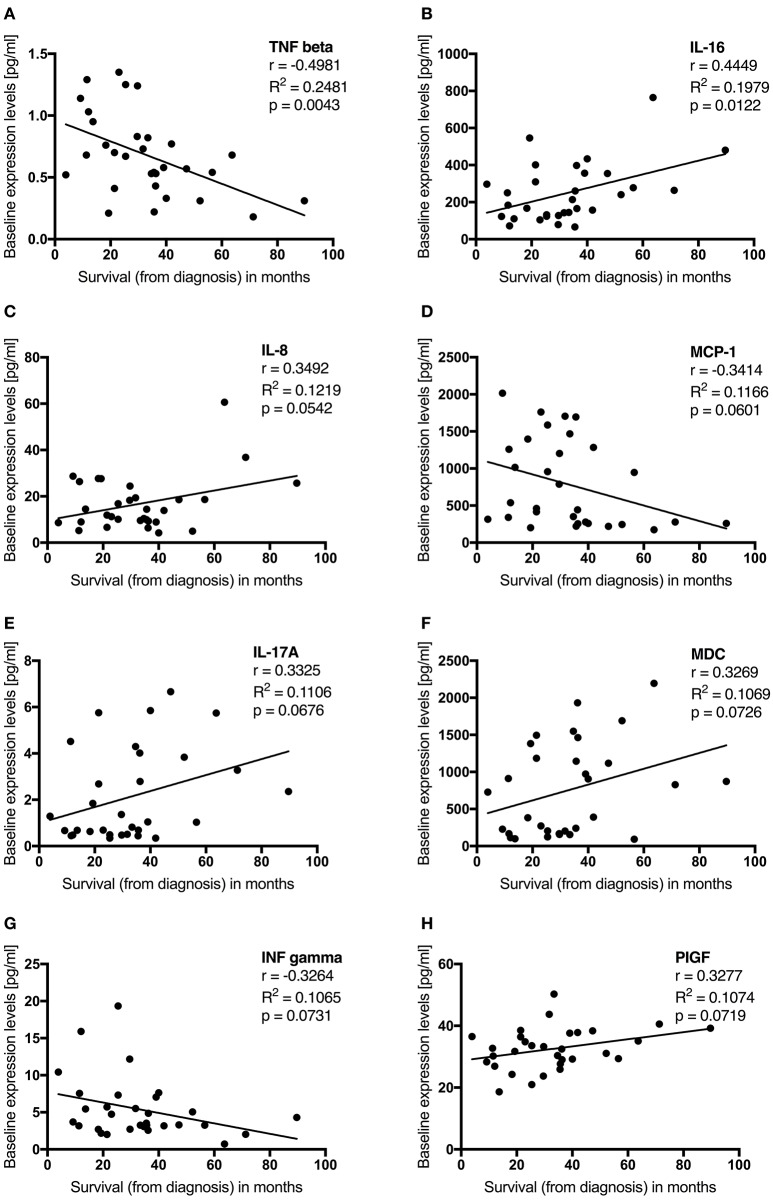
**(A–D)** Baseline cytokine levels are associated with survival in G-CSF treated ALS patients. Plotted are initial cytokine levels of TNF beta **(A)**, IL-16 **(B)**, IL-8 **(C)**, MCP-1 **(D)**, IL-17A **(E)**, MDC **(F)**, ING gamma **(G)**, and PIGF **(H)** in pg/ml before first G-CSF application in 31 patients. Survival was assessed in months from diagnosis and censored upon data admission in living patients (*n* = 7). Displayed is Pearson r, the coefficient of determination (*R*^2^), *p*-value (two-tailed) significant at *p* < 0.05, trend at *p* < 0.1.

Survival in months from diagnosis was negatively correlated with baseline serum levels of the cytokine TNF-beta. MCP-1 and INF-gamma were, as a trend, negatively correlated with survival as well. IL-16 baseline levels displayed a positive correlation with survival. MDC, IL-8, IL-17A, and PIGF were, as a trend, positively correlated with survival (Table 2, Figure 5). We then dichotomized G-CSF treated patients according to their survival of either more or less than 30 months from diagnosis, and analyzed cytokines at baseline. Patients who survived longer than 30 months from diagnosis had significantly higher baseline levels of MDC and Tie-2. For IL-16, IL-17A, and PIGF we found similar trends. On the other hand, there were significantly higher baseline levels of TNF-beta and IL-7 in patients who survived less than 30 months from diagnosis. TNF-alpha and MCP-1 displayed similar trends. However, when correcting the cytokine comparisons in long and short survival for multiple testing, none of these findings remained significant [as assessed by the FDR-adjusted *p*-values (*q*-values) in Table 2].

**Table 2:** Cytokine levels at baseline in relation to survival.

**Cytokine**	**Level in long survival**	**Correlation**			**Comparison (*****t*****-test)**			
		***r*-value**	***R*^2^-value**	***p*-value**	**Median long survival**	**Median short survival**	***p*-value**	***q*-value**
**ANTI-INFLAMMATORY**								
MDC	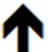	0.3269	0.1069	0.0726	939	227	**0.0494**	0.3088
**PRO-INFLAMMATORY**								
TNF-ß	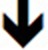	−0.4981	0.2481	**0.0043**	0.535	0.830	**0.0038**	0.1254
IL-7	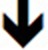	–	–	–	17	27	**0.0171**	0.2640
TNF-α	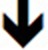	–	–	–	2.5	3.0	0.0638	0.3088
MCP-1	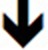	−0.3414	0.1166	0.0601	278	957	0.0544	0.3088
INF-γ	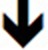	−0.3264	0.1065	0.0731	–	–	–	–
IL-16	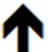	0.4449	0.1979	**0.0122**	262	133	0.0655	0.3088
IL-8	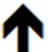	0.3492	0.1219	0.0542	–	–	–	–
IL-17A	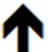	0.3749	0.1406	0.0710	2.58	0.68	0.0912	0.3421
**ANGIOGENESIS**								
Tie-2	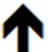	–	–	–	5762	4492	**0.0240**	0.2640
PIGF	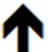	0.3277	0.1074	0.0719	33.8	31.7	0.0933	0.3421

*Cytokine levels in pg/ml before first G-CSF application in ALS patients. Arrows indicate cytokine levels in patients with long compared to short survival. Then cytokine levels at baseline were correlated with survival. Next, Mann-Whitney test was calculated to assess differences in baseline cytokine levels in patients with survival longer or shorter than 30 months from diagnosis. Number of patients at baseline: 31 (16 long survival). Significance is indicated by bold marking when p < 0.05 (two-tailed p-value), trend when p < 0.1. T-tests were corrected for multiple testing by FDR-adjusted p-values (q-values), discovery is indicated by q < 0.1*.

### G-CSF treatment modulated serum cytokine levels of ALS patients over time

The direct effects of G-CSF on cytokine levels were evaluated by comparing cytokine levels 2 days prior to and 1 day after ongoing 5-day G-CSF application in a subgroup of patients allowing this analysis. These immediate effects were determined at three different time points (baseline, 3 and 6 months after treatment initiation). Due to individual application modes, 5-day G-CSF applications with corresponding blood samples were available for 18 patients at baseline, for 17 patients at 3 months, and for 14 patients at 6 months of ongoing G-CSF treatment.

We found G-CSF to have an immediate effect on the level of various cytokines (Table 3, Figure S3). The serum level of IL-10 increased after 5 days of G-CSF treatment at baseline, 3 months and 6 months compared to its respective level before G-CSF application, however, at 3 months only as a trend. The levels of IL-16, Tie-2, TNF-alpha, MIP1-beta, IL-15, IP-10, VCAM, ICAM-1, and of Flt-1 were significantly increased after G-CSF treatment at all above-mentioned time points. The levels of TARC, IL-7, INF-gamma, and MCP-1 were decreased at all above-mentioned time points. There was an increase in SAA, IL-12/IL-23p40, CRP, and VEGF-A levels after G-CSF at baseline and 6 months, the latter at 6 months only as a trend. The levels of VEGF-C and PIGF were increased after G-CSF at 6 months, that of PIGF also at 3 months as a trend. There was a decrease of Eotaxin-1, Eotaxin-3 and VEGF-D after G-CSF application at baseline and 3 months. TNF-beta was decreased at baseline, at 6 months by a trend. MCP-4 was decreased at 6 months, at baseline by a trend. The level of bFGF was decreased after G-CSF application at 3 months and 6 months.

**Table 3:** Cytokine levels before and after G-CSF treatment at different time points.

**Cytokine**	**Direction**	**Treatment start**			**3 months**			**6 months**		
		**Fold change d0-d7**	***p*-value**	***q*-value**	**Fold change d0-d7**	***p*-value**	***q*-value**	**Fold change d0-d7**	***p*-value**	***q*-value**
**ANTI-INFLAMMATORY**										
IL-10	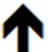	1.95	**0.0016**	**0.0018**	1.24	0.0856	0.0371	2.02	**0.0004**	**0.0007**
**PRO-INFLAMMATORY**										
TNF-ß	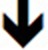	0.83	**0.0208**	**0.0112**	–	–	–	0.85	0.0591	0.0268
INF-γ	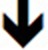	0.81	**0.0214**	**0.0113**	0.78	**0.0182**	**0.0104**	0.62	**0.0009**	**0.0014**
IL-7	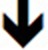	0.66	**0.0003**	**0.0006**	0.80	**0.0011**	**0.0014**	0.80	**0.0107**	**0.0075**
MCP-1	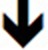	0.83	**0.0120**	**0.0080**	0.77	**0.0007**	**0.0012**	0.79	**0.0107**	**0.0075**
MCP-4	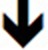	0.94	0.0599	0.0268	0.92	**0.0150**	**0.0089**	–	–	–
TARC	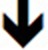	0.87	**0.0304**	**0.0148**	0.86	**0.0032**	**0.0030**	0.80	**0.0166**	**0.0097**
Eotaxin-1	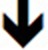	0.88	**0.0139**	**0.0085**	0.93	**0.0079**	**0.0059**	–	–	–
Eotaxin-3	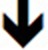	0.83	**0.0034**	**0.0030**	0.78	**0.0034**	**0.0030**	–	–	–
CRP	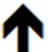	4.45	**0.0010**	**0.0014**	–	–	–	3.99	**0.0085**	**0.0062**
SAA	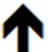	2.96	**0.0008**	**0.0013**	–	–	–	2.03	**0.0353**	**0.0168**
TNF-α	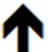	1.77	<**0.0001**	**0.0003**	1.50	**0.0046**	**0.0038**	1.79	**0.0004**	**0.0007**
IP-10	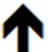	1.50	**0.0002**	**0.0004**	1.34	**0.0067**	**0.0052**	1.40	**0.0134**	**0.0085**
IL-15	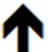	1.14	**0.0022**	**0.0022**	1.24	**0.0208**	**0.0112**	1.24	**0.0016**	**0.0018**
IL-12/IL-23p40	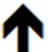	1.24	**0.0047**	**0.0038**	–	–	–	1.19	**0.0052**	**0.0041**
IL-16	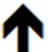	3.14	<**0.0001**	**0.0003**	3.66	**0.0011**	**0.0014**	3.78	**0.0002**	**0.0004**
MIP1-ß	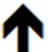	3.38	<**0.0001**	**0.0003**	4.63	**0.0013**	**0.0015**	2.91	**0.0001**	**0.0003**
**ANGIOGENESIS**										
VEGF-A	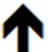	1.39	**0.0010**	**0.0014**	–	–	–	1.32	0.0580	0.0268
Tie-2	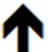	1.27	<**0.0001**	**0.0003**	1.18	**0.0032**	**0.0030**	1.19	**0.0134**	**0.0085**
Flt-1	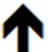	1.45	<**0.0001**	**0.0003**	1.32	**0.0026**	**0.0026**	1.33	**0.0001**	**0.0003**
PIGF	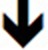	–	–	–	–	–	–	1.14	**0.0203**	**0.0112**
VEGF-C	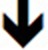	–	–	–		0.0984	0.0420	0.86	**0.0017**	**0.0017**
VEGF-D	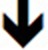	0.88	**0.0139**	**0.0085**	0.94	**0.0110**	**0.0075**	–	–	–
bFGF	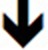	–	–	–	0.72	**0.0232**	**0.0121**	0.81	**0.0040**	**0.0035**
**VASCULAR INJURY**										
VCAM	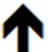	1.40	<**0.0001**	**0.0003**	1.28	**0.0267**	**0.0134**	1.41	**0.0001**	**0.0003**
ICAM-1	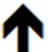	1.30	<**0.0001**	**0.0003**	1.23	**0.0305**	**0.0148**	1.31	**0.0001**	**0.0003**

*Paired Wilcoxon t-test. Arrows indicate direction, and fold change gives effect size of cytokine modulation when comparing respective levels 2 days before (d0) and 1 day after (d7) daily application of G-CSF over 5 days. Number of evaluable patients at baseline: 18, at 3 months: 17, and at 6 months: 14. Significance is indicated by bold marking when p < 0.05 (two-tailed p-value), trend when p < 0.1. T-tests were corrected for multiple testing by FDR-adjusted p-values (q-values), discovery is indicated by q < 0.1. Non-significant and non-trend findings are marked by -*.

## Discussion

### Our main findings

Long term and individually adapted off-label treatment with G-CSF in 36 ALS patients was well tolerated and safe. The number of G-CSF-mobilized hematopoietic stem cells, as measured by AUC, was associated with longer survival. Initial levels of serum cytokines, such as MDC, TNF-beta, IL-7, IL-16, and Tie-2 were significantly associated with survival, indicating the potential of prognostic application of these immune markers in G-CSF treated ALS patients. Continued application of G-CSF led to persistent alterations in various serum cytokines and ongoing measurements revealed the multifaceted effects of G-CSF.

### ALS as a neuroinflammatory disease

ALS has been recognized as a multifactorial disease. Neurodegenerative processes trigger neuroinflammation and vice versa. Neuroinflammation with microglial activation, infiltration of peripheral immune cells into the CNS, and alterations in cytokine levels are pathological features in ALS. Cytokines are mediators of the immune communication that may cross the blood-brain barrier (BBB) and provide a mechanism by which the peripheral immune system may directly influence the CNS (30). In a recent study, we demonstrated a pro-inflammatory immune response with elevated inflammatory cytokines both in serum during disease and post-mortem in the CNS of ALS patients (31). However, immune response in ALS cannot be clearly dichotomized to a purely pro- or anti-inflammatory state, as cytokines are often pleiotropic, and their function may change over time and depend on concentration and specific disease context. Possibly, cytokine response in early ALS may be an attempt to restore homeostatic balance, whereas chronic exposure to pro-inflammatory cytokines might lead to cell destruction and loss of neuronal function. The latter supports a self-sustaining inflammatory process and possibly accelerates disease progression (7). Neuroinflammation and systemic inflammatory stimuli with their influence upon the CNS offer targets for therapeutic intervention in ALS (32). Analysis of peripheral blood is a feasible alternative for ongoing measurements of immune mediated and pathophysiological relevant parameters (33).

### G-CSF in ALS

ALS is a multifactorial disease. Targeting common pathologic features such as neuro-inflammation and -degeneration may thus be beneficial for all ALS patients. Although G-CSF is an established, well-tolerated and safe growth factor for mobilization of hematopoietic stem and precursor cells (34), there is accumulating evidence that it is also a potent modulator of multiple CNS functions relevant to ALS (13). G-CSF modulates the immune response (35), it promotes anti-inflammatory and decreases pro-inflammatory mediators (36). Small clinical trials with G-CSF in ALS patients have delivered inconclusive results. Treatment with G-CSF was associated with a decrease in pro-inflammatory cytokine levels in serum and cerebrospinal fluid (CSF) of ALS patients (26), and minor benefits were detected by neuroimaging (27). But promising evidence for efficacy of G-CSF in ALS animal models has not yet been translated to ALS patients. It seems likely that a successful clinical translation requires higher dose, more frequent application and longer exposure to G-CSF as well as extended observation times (37). The latter is of crucial importance when aiming at structural and functional improvements or support of neurogenesis.

### G-CSF treatment in ALS is safe and well tolerated

Application of G-CSF in oncological indications is usually limited to treatment cycles, and the only clinical experience with life-long G-CSF therapy has accumulated with patients suffering from severe congenital neutropenia and cyclic neutropenia (38, 39). To our knowledge, we first reported on long-term G-CSF treatment in a CNS indication (25). We found G-CSF application to be generally well tolerated in ALS patients, with mild to moderate bone pain and leukocytosis after G-CSF applications as frequent minor adverse events. As this was off-label, experimental treatment of individual ALS patients, we had no control group for assessment of survival. If we only observe *deceased* patients and leave those still alive out, then the mean survival of these 26 patients at 25.5 months from diagnosis indicates no harm by G-CSF in ALS.

### Stem cell mobilization is efficient and associated with longer survival in G-CSF treated ALS patients

G-CSF is a well-known mobilizer of hematopoietic stem cells (8, 9). In all patients treated with G-CSF for five consecutive days, G-CSF increased mobilization of hematopoietic stem cells (CD34^+^ and CD34^+^ CD38^−^) into the peripheral blood. Interestingly, we found an association between stem cell mobilization and survival. Patients who survived longer than 30 months from diagnosis mobilized more CD34^+^CD38^−^ hematopoietic stem cells than patients with shorter survival, as measured by Area Under the Curve after G-CSF treatment up to 1 year (Figure 4). Higher levels of circulating hematopoietic stem cells are associated with better clinical outcome and less structural damage after intracerebral hemorrhage in humans (40). The mechanism of how hematopoietic stem cells may contribute to neurodegenerative disease is yet unclear. Migration and subsequent trans-differentiation of bone marrow derived cells within the CNS is controversially discussed (18). However, G-CSF increases the number of hematopoietic stem cells translocated to the damaged CNS (16, 17). There, hematopoietic stem cells modulate the immune system, they may interact with local cells, and produce neurotrophic factors, which promote growth of neural progenitors and survival (17, 18). A recent study in mice exposed to cranial irradiation demonstrated that G-CSF augments neurogenesis; bone marrow derived G-CSF-responsive cells migrate to the CNS, where they express macrophage and microglia phenotypes. The authors found that G-CSF treatment led to an improved functional outcome, thus arguing for the neuroprotective mechanisms of G-CSF on brain repair (15). Human studies have demonstrated G-CSF to directly affect monocytes and to modulate monocyte cytokine secretion toward an anti-inflammatory polarization (41). A recent study applying G-CSF in healthy humans described expansion of a mature variant monocyte subtype displaying strong immunosuppressive properties (42). Next to neural cells, also neural stem cells have G-CSF receptors and G-CSF treatment induces a neural phenotype of these cells (12). Effects of G-CSF on hematopoietic stem cells may therefore serve as a proxy for biological cellular activity of G-CSF on neural cells.

### Cytokine levels are associated to survival and affected by G-CSF

Neuroinflammation contributes to the pathogenesis of ALS (3). Apart from CNS inflammation, peripheral cytokines and other inflammatory markers are affected in ALS, and cytokine levels may serve as biomarkers (43). We found that different cytokines at baseline were correlated with survival (Table 2, Figure 5). When dichotomizing patients depending on individual survival being longer or shorter than 30 months from diagnosis, we detected different peripheral cytokine levels at baseline (Table 2). In general, five-day treatment courses with G-CSF exerted immediate effects on cytokine levels and were able to partly counteract the harmful immune response in ALS (Table 3, Figure S3).

The initial levels of 11 cytokines were associated with survival, of which 8 were altered by G-CSF application. However, the correlation models, as indicated by the rather low *R*^2^-values, could only explain smaller parts of the variance. Even though the cytokine comparisons in long and short survival did not withstand correction for multiple testing, we decided to explore the findings because they might help to generate hypotheses for further studies and show biologically important findings in spite of the small number of patients tested. Moreover, G-CSF led to change in many inflammatory cytokines, as well as cytokines involved in angiogenesis and vascular injury, of which all significant changes remained so after testing for multiple comparison (Tables 2, 3).

Initial TNF-beta (LTA, lymphotoxin-alpha) levels negatively correlated with survival and were found at higher levels in shorter surviving ALS patients upon treatment initiation. G-CSF application led to reduction in TNF-beta, a pro-inflammatory cytokine and common cell death effector found to be increased in ALS sera (31). TNF-alpha was borderline increased in patients with shorter survival (trend) and G-CSF led to an increase in its serum levels. TNF-alpha is elevated in ALS (31, 43–46) and correlates with disease duration (47). But its role in ALS in unclear and the two TNF-alpha receptors, either associated with inflammation or neuroprotection, have opposing effects concerning survival in ALS (48). Dependent on subtype and context, activation can lead to neuroprotection and neurogenesis (49), reduced oxidative stress (50) and glutamate excitotoxicity (51). An increased occurrence of ALS after long-term use of TNF-alpha inhibitors in rheumatic arthritis, is suggested to be a consequence of deficient TNF-alpha mediated neuronal protection (52). Higher initial levels of IL-7 were associated with shorter survival, and reduced after ongoing treatment with G-CSF. IL-7 is considered a pro-inflammatory cytokine, and is increased in CSF (53) and serum (31) of ALS patients. MCP-1 (CCL2) was borderline correlated (trend) with shorter survival of ALS patients. We confirmed a reduction of MCP-1 levels in ALS after treatment with G-CSF (26). MCP-1 is a prominent pro-inflammatory cytokine that can enhance microglial recruitment to the CNS after injury, which may exacerbate ALS progression (54). MCP-1 correlates with faster disease progression (55) and ALS patients have elevated MCP-1 serum levels (31, 55, 56) and increased protein expression within spinal cord (31). INF-gamma was borderline negatively correlated with survival in our patients (trend). As known from healthy donors (57), INF-gamma levels were decreased by G-CSF. As a hallmark of proinflammatory cells, INF-gamma is proposed to contribute to motor neuron death in ALS (58). ALS patients have higher INF-gamma serum levels (47, 55, 59), that correlate with disease progression (47, 59) and shorter survival (55).

On the other hand, the pro-inflammatory marker IL-16 was positively correlated with survival and increased after G-CSF application. IL-16 also holds an immunomodulatory role by expansion of regulatory T cells (Treg) (60), that at lower levels in ALS, are associated with rapid disease progression and shorter survival (61). Thus, G-CSF related increase in IL-16 might be beneficial for ALS patients. Another pro-inflammatory cytokine, IL-17A, was borderline correlated with longer survival (trend) but not altered by G-CSF treatment. IL-17A has been reported elevated in serum (55, 62, 63) and CSF (64) of ALS patients. After G-CSF treatment, Chió et al. found a reduction of IL-17A in the CSF, but not in serum of ALS patients (26). IL-8 was borderline correlated with longer survival (trend), and not altered by G-CSF treatment. IL-8 is produced by several cells in response to inflammation, and higher plasma (44) and CSF levels (65) are known in ALS. MDC (CCL22) was associated with longer survival, however, not modulated by G-CSF treatment. MDC is an anti-inflammatory cytokine, and consistent with a proposed protective effect, ALS patients have lower MDC protein expression in the spinal cord (31). Further, angiogenic factors, such as Tie-2 and PIGF were associated with survival. Tie-2 was elevated in longer surviving patients and G-CSF led to an increase in it's serum levels. Angiogenesis is mediated by the angiopoietin-1/Tie-2 system (66), and stimulation of angiogenesis by another pro-angiogenic factor, VEGF, is found to increase neurogenesis (19). G-CSF treatment led to an increase in PIGF, and PIGF was as a trend both correlated with survival and elevated in longer surviving patients. PIGF supports angiogenesis (67), and may be a marker for the angiogenic niche.

The following 18 cytokines were significantly altered by G-CSF, however, not associated with survival. As known from healthy donors (68), IL-10 was markedly increased after G-CSF treatment. This anti-inflammatory cytokine is elevated in ALS-patients with mild symptoms or slow progression (53). G-CSF application led to reduced systemic levels of the pro-inflammatory cytokines MCP-4 (CCL13), TARC, Eotaxin-1 (CCL11), and Eotaxin-3 (CCL26). MCP-4 (31, 65), TARC (31) and Eotaxin-1 (65) are elevated in ALS serum. The latter is further associated with Alzheimer's dementia (69), aging and inhibition of neurogenesis in mice (70). We also noticed increase in levels of the pro-inflammatory cytokines CRP, SAA, IP-10 (CXCL10), IL-15, IL-12/IL-23p40, and MIP1-beta after G-CSF application. The acute-phase proteins CRP and SAA have been described as elevated in ALS patients (31, 71). IP-10 is negatively correlated with disease progression rate (72) and increase after G-CSF treatment has been described (26). IL-15 (31, 55, 73) and MIP1-beta (31) are elevated in serum of ALS patients. MIP1-beta shares receptor (CCR5) with MIP-alpha, which is elevated and considered neuroprotective in ALS (74). MIP-1 beta is negatively correlated with disease severity and progression rate, and thus might exert neuroprotective effects in ALS (72). IL-12/IL-23p40 describes the p40 subunit shared by the cytokines IL-12 and IL-23, and is considered a pro-inflammatory marker. However, we noted no increase in cytokines induced by IL-12/IL-23p40, such as INF-gamma and IL-17A, after G-CSF treatment. Aside from neuroinflammation, impaired neurotrophic support is a hallmark of ALS. Levels of VEGF-A and Flt-1 were increased, whereas VEGF-C, VEGF-D, and bFGF levels were decreased after G-CSF application. VEGF-A and bFGF, two common neurotrophic and possibly protective factors in ALS (55), are both increased in ALS CSF (64). Further, VEGF-A supports neurogenesis and neural development and is an attractant for HSPC that has been associated with longer survival in ALS (55). We found an increase in ICAM-1 and VCAM-1 after G-CSF treatment. At the vascular endothelium these cellular adhesion molecules are involved in leukocyte transport (75), but their role in ALS is unclear.

In ALS, a short time delay for diagnosis is associated with inferior prognosis as these patients are likely to have a more aggressive disease (76). Accordingly, we observed a briefer latency between diagnosis and treatment initiation in patients with shorter survival, which might reflect a more rapid progression of disease in these patients. Hence, longer surviving patients presumably initiated treatment at a later pathophysiological stage of their disease. This might offer an explanation for the fact that levels of some pro-inflammatory cytokines such as IL-16, IL-17A, and IL-8 were associated with longer survival. However, the role of inflammatory markers in ALS is unclear and our findings may also indicate that inflammation does not only negatively impact the disease (71). The remaining relation between cytokines and survival seen in our cohort highlights the importance of these markers in predicting individual survival. Thus, different cytokines may be used as biomarkers for initial patient stratification, predicting later clinical course, monitoring treatment response and progression of disease.

Possible direct effects of G-CSF upon the CNS were not assessed, as we did not obtain post-mortem analysis of deceased patients. Neuroimaging studies conducted on our G-CSF treated patient cohort (77) did not directly address possible G-CSF related structural effects—we also had no patient control group without G-CSF treatment. One indirect mode of action by which G-CSF exerts neuroprotective effects may be through polarization of the immune system toward an anti-inflammatory state (13). We observed an increase in anti-inflammatory cytokines and neurotrophic factors as well as a decrease in pro-inflammatory cytokines. However, we also captured an increase in some pro-inflammatory cytokines, which might be due to the pleiotropic effects of G-CSF and possibly reflect an unspecific cytokine reaction after application. Overall, the effects of G-CSF on peripheral cytokine levels and ALS appear to be versatile and should be assessed in a prospective clinical study.

### Strengths and limitations

This retrospective analysis has several limitations. Firstly, we have not conducted a controlled clinical trial and thus, there was no placebo-arm. Rather, the aim of the intervention was to offer individual ALS patients a potentially beneficial off-label treatment with G-CSF. Evaluation of respiratory function was driven by clinical indication and not systematically assessed. Hence, we did not regularly screen for respiratory deficits upon treatment initiation. The same applied to assessment of cognitive function. In addition, we did not systematically analyze for ALS-specific gene mutations. Such factors have predictive value concerning prognosis (78), the lack of initial screening of respiratory and cognitive function as well as genetic background might impede interpretation of the data. Given the objective of evaluating safety of G-CSF and the absence of a control group, in this paper we assessed survival from time of diagnosis, and not from treatment initiation. The latency between symptom onset and diagnosis was not assessed in this report. This is a limitation, as quantification of diagnostic delay - being associated with longer survival (78), could have offered prognostic implications. During the experimental treatment, patients were routinely seen on an outpatient basis to monitor safety and blood samples were regularly obtained. This enabled a dynamic observation of alterations in neuroinflammation due to ALS disease and treatment with G-CSF over time. However, with only 36 G-CSF treated patients caution should be applied in trying to generalize our findings. Moreover, application and dosing schemes for G-CSF treatment were decided upon on an individual patient level and thus complicated the establishment of dose-effect relationships. When we analyze cytokine levels upon treatment initiation in our patient cohort retrospectively, we have to take into account that these patients differ concerning covariant factors such as age, gender, bulbar vs. spinal-onset, and functional status (ALSFRS-R). Given the small number of patients treated with G-CSF, a statistical evaluation of the predictive value of these subpopulations was not reasonable. There was also great heterogeneity in the latency between time of diagnosis and treatment initiation. Cytokine levels alter during progression of disease. Altogether, these aspects lead to a reduced statistical power, which may also provide an explanation for the variation and modest correlation seen between initial cytokine levels and survival. Moreover, we found that cytokine comparisons in long and short surviving patients did not withstand correction for multiple testing. These signals may be of biological relevance, as they were detected in spite of a small number of patients and great disease heterogeneity, and thus may assist in hypothesis generation for future studies.

## Conclusion

Altogether, we found that long term G-CSF treatment is feasible and safe for ALS patients. G-CSF efficiently mobilized hematopoietic stem cell into peripheral blood, and the amount of mobilized stem cells was associated with longer survival. Thus, stem cell mobilization could be a potential biomarker to monitor treatment response to G-CSF. Peripheral cytokines are relevant in the course of disease in ALS. We identified TNF-beta, MDC, IL-16, IL-7, and Tie-2 as cytokines whose baseline levels may predict G-CSF treatment response and survival. Additionally, long term G-CSF treatment led to sustained alterations of multiple cytokines in peripheral blood. Thus, cytokines represent potential biomarkers for survival prediction and for early monitoring of G-CSF treatment in ALS, all of which need further validation in a prospective controlled randomized trial.

## Author contributions

SJ care for ALS patients, conception of intervention, analysis, interpretation, wrote the manuscript. UB clinical responsibility for intervention. UB, WS-M, and VS care for ALS patients, conception of intervention and analysis, revision of the manuscript. T-HB conception of intervention and analysis, revision of the manuscript. BB contribution to analysis, revision of the manuscript. A-LM care for ALS patients, revision of the manuscript. TK organization of care and disposition of patient material. SP and EW performed cytokine experiments, revision of the manuscript. SI and JG performed and analyzed HSPC experiments, revision of the manuscript. AS and WH revision of the manuscript.

### Conflict of interest statement

UB holds patents for clinical application of G-CSF in ALS, Orphan Drug Status is granted for EU and US by EMA and FDA–all within NeuroVision Pharma GmbH, Murnau, Germany. The remaining authors declare that the research was conducted in the absence of any commercial or financial relationships that could be construed as a potential conflict of interest.
