# Supplementary material for: Biomarker Supervised G-CSF (Filgrastim) Response in ALS Patients
Source: Front Neurol. 2018 Nov 26;9:971. doi: 10.3389/fneur.2018.00971 (PMC6275232; doi:10.3389/fneur.2018.00971)
Supplement: Supplementary file 1 [file Table_1.pdf]

ALS patient		1st month	2nd month	3rd month	4th month	5th month	6th month
1	Dose	150	150	150	150	150	150
	Application	A	A	A	A	A	A
2	Dose	150	150	150	240	240	240
	Application	A	A	A	A	A	A
3	Dose	150	150	150	150		
	Application	A	A	A	A		
4	Dose	150	150	150	150		
	Application	A	A	A	A		
5	Dose	150	150	240	240	240	240
	Application	A	A	A	A	A	A
6	Dose	150	150	150	150	150	150
	Application	A	A	A	A	A	A
7	Dose	240	240	240	240	240	240
	Application	E	E	E	E	E	E
8	Dose	150	150	150	150	150	150
	Application	A	A	A	A	A	A
9	Dose	150	150	90	90	150	150
	Application	A	A	D	D	A	A
10	Dose	150	150	150	150	150	240
	Application	A	A	A	A	A	A
11	Dose	150	150	240	300	300	300
	Application	A	A	A	A	A	A
12	Dose	240	300	300	300	300	300
	Application	A	A	A	A	A	A
13	Dose	150	150	150	150	150	150
	Application	A	A	A	A	A	A
14	Dose	192	192	192	192	192	192
	Application	E	E	E	E	E	E
15	Dose	192	192	192	192	192	192
	Application	E	E	E	E	E	E
16	Dose	150	300				
	Application	A	A				
17	Dose	192	192	192	192	192	192
	Application	E	E	E	E	E	E
18	Dose	240	240	240	240	240	240
	Application	E	E	E	E	E	E

ALS patient		1st month	2nd month	3rd month	4th month	5th month	6th month
19	Dose	150	150	150	150	150	150
	Application	A	A	A	A	A	A
20	Dose	240	192	192	192	192	192
	Application	A	E	E	E	E	E
21	Dose	150	150	150	150	150	150
	Application	A	A	A	A	A	A
22	Dose	240	240	480	480	480	480
	Application	A	A	A	B	B	B
23	Dose	150	300	300	300		
	Application	A	A	A	A		
24	Dose	150	300	300	480	510	510
	Application	A	B	B	B	C	C
25	Dose	240	300	300	300	300	480
	Application	A	B	B	B	B	B
26	Dose	240	480	480	480	480	900
	Application	A	B	B	B	B	F
27	Dose	240	240	480	720	720	720
	Application	A	A	B	F	F	F
28	Dose	480	480	480	480	480	480
	Application	B	B	B	B	B	B
29	Dose	150	450	450	720	720	720
	Application	A	F	F	F	F	F
30	Dose	450	450	450	720	720	720
	Application	F	F	F	F	F	F
31	Dose	240	720	720	720	720	720
	Application	A	F	F	F	F	F
32	Dose	450	720	720	720	720	720
	Application	F	F	F	F	F	F
33	Dose	450	450	450	720	720	720
	Application	F	F	F	F	F	F
34	Dose	480	450	450	720	1440	1440
	Application	A	F	F	F	F	F
35	Dose	450	450	720	720	1056	1056
	Application	F	G	G	F	G	G
36	Dose	300	480	480	720	1296	1296
	Application	B	B	B	F	G	G

Table S1: Dose and application over the first 6 months of G-CSF treatment. Illustration of the heterogeneity in treatment with individual doses and application modes, and change within single patients over time. Doses of G-CSF are given as cumulative monthly dose (Mio IU). Application with five-day treatment is indicated by a darker grey color and application on single days by a brighter grey color. Application termed “A”: G-CSF once or twice á day over five consecutive days á month. “B”: treatment as in “A” repeated once. “C”: treatment as in “B” with G-CSF application every second day between the five-day treatments. “D”: G-CSF on 3 days á month. “E”: G-CSF once or twice á day on a single day á week. “F”: G-CSF every second day. “G”: other combination of G-CSF on single days á week.
