# Supplementary material for: Biomarker Supervised G-CSF (Filgrastim) Response in ALS Patients
Source: Front Neurol. 2018 Nov 26;9:971. doi: 10.3389/fneur.2018.00971 (PMC6275232; doi:10.3389/fneur.2018.00971)
Supplement: Supplementary file 2 [file Image_1.pdf]

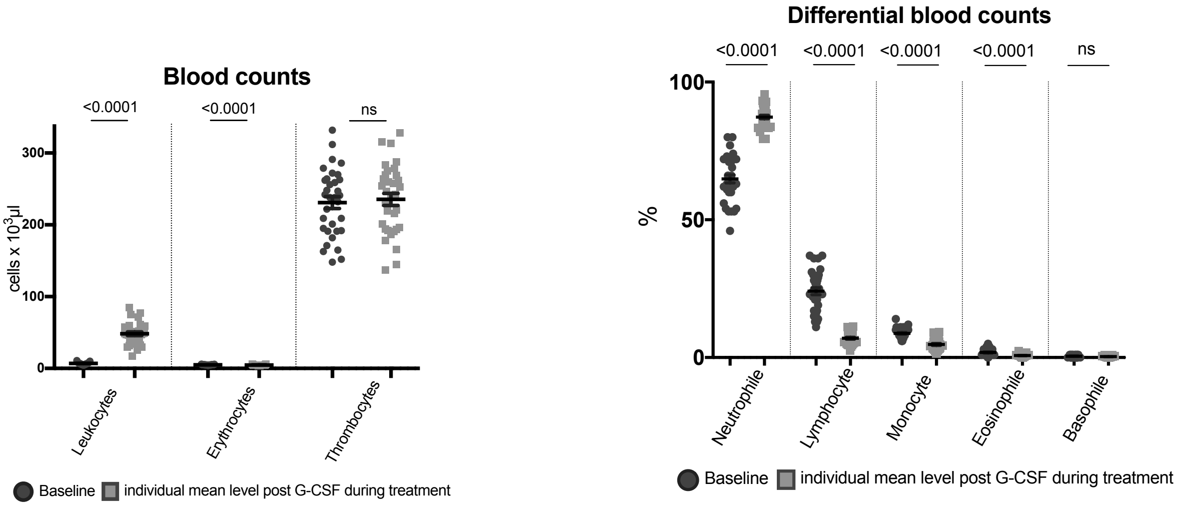


**Figure S1: Effect of G-CSF on blood counts** over time. Baseline indicates measurement before first G-CSF application, and is compared to individual mean level after G-CSF application over time (up to closure of data admission) in 33 patients (3 patients without baseline levels). Data are presented as scatter dot plot with mean + SEM. Paired t test, p-value (two-tailed) significant at $p<0.05$.

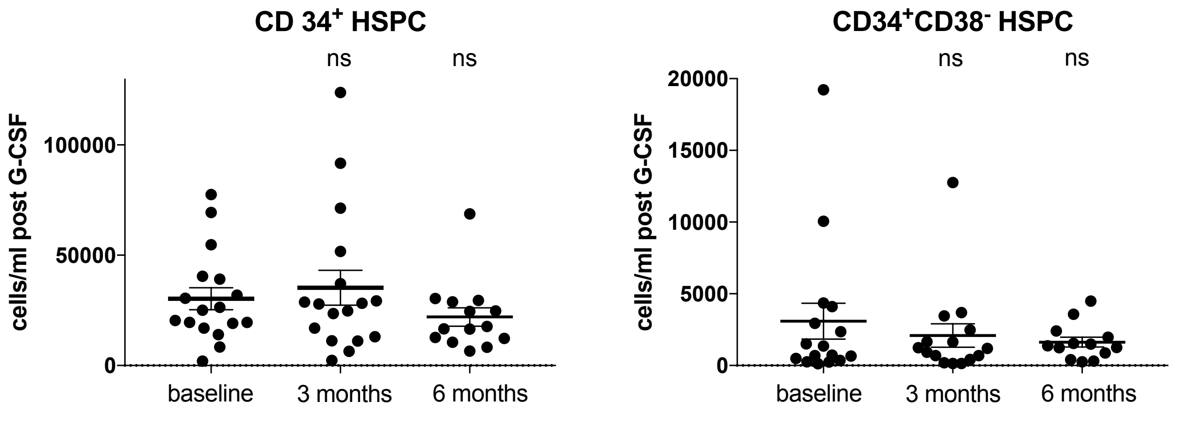


**Figure S2: Hematopoietic stem cells (HSPC) after G-CSF** at three time points in 17, 17 and 14 patients ($CD34^+$) and 16, 15 and 13 patients ($CD34^+CD38^-$) on five day treatment. Comparison between baseline post G-CSF level and levels at 3 and 6 months by Wilcoxon paired t test. Data are presented as scatter dot plot with mean + SEM. Paired t test, p-value (two-tailed) significant at $p<0.05$.

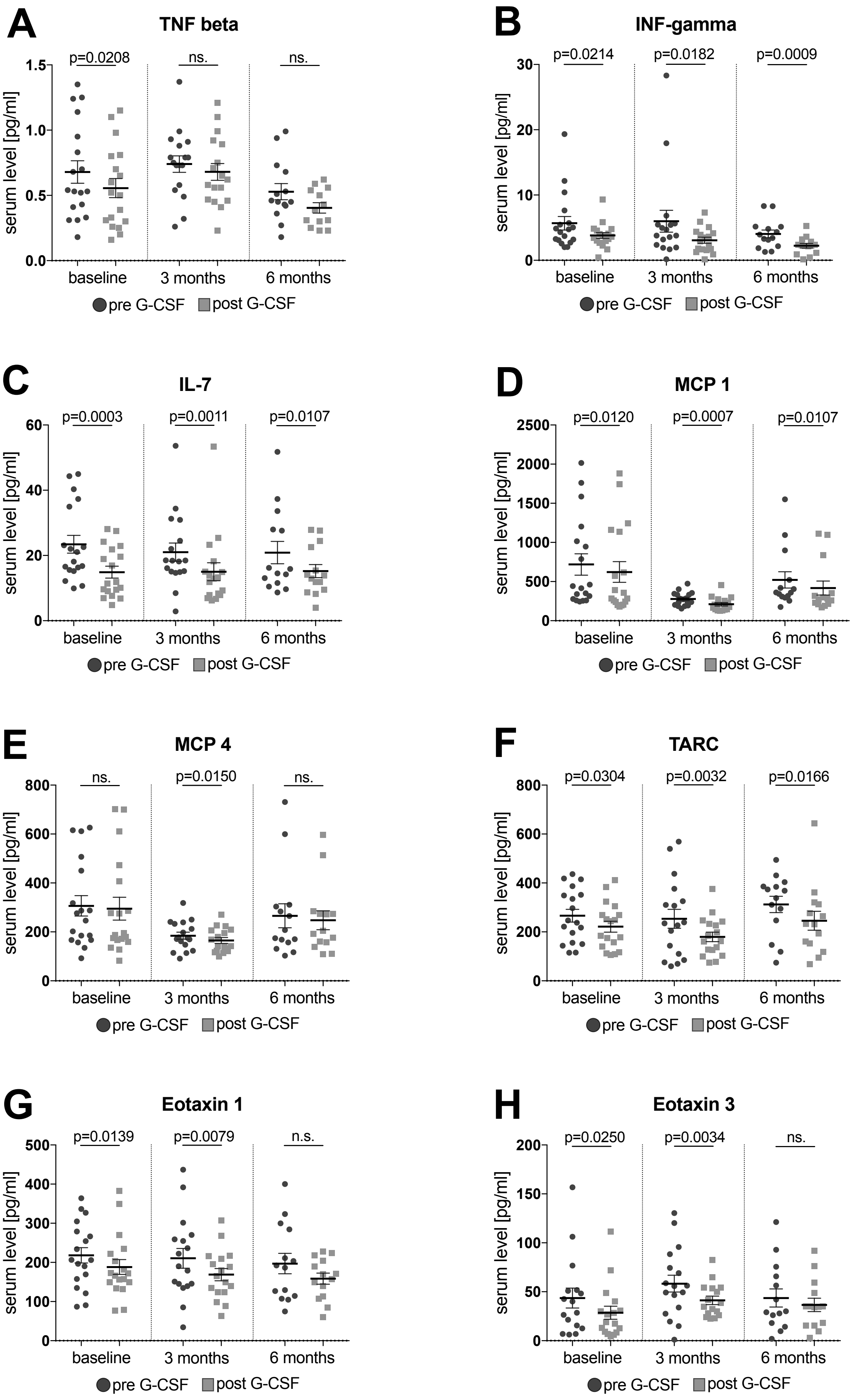
A
TNF beta
p=0.0208
ns.
ns.
serum level [pg/ml]
1.5
1.0
0.5
0.0
baseline
3 months
6 months
pre G-CSF
post G-CSF
B
INF-gamma
p=0.0214
p=0.0182
p=0.0009
serum level [pg/ml]
30
20
10
0
baseline
3 months
6 months
pre G-CSF
post G-CSF
C
IL-7
p=0.0003
p=0.0011
p=0.0107
serum level [pg/ml]
60
40
20
0
baseline
3 months
6 months
pre G-CSF
post G-CSF
D
MCP 1
p=0.0120
p=0.0007
p=0.0107
serum level [pg/ml]
2500
2000
1500
1000
500
0
baseline
3 months
6 months
pre G-CSF
post G-CSF
E
MCP 4
ns.
p=0.0150
ns.
serum level [pg/ml]
800
600
400
200
0
baseline
3 months
6 months
pre G-CSF
post G-CSF
F
TARC
p=0.0304
p=0.0032
p=0.0166
serum level [pg/ml]
800
600
400
200
0
baseline
3 months
6 months
pre G-CSF
post G-CSF
G
Eotaxin 1
p=0.0139
p=0.0079
n.s.
serum level [pg/ml]
500
400
300
200
100
0
baseline
3 months
6 months
pre G-CSF
post G-CSF
H
Eotaxin 3
p=0.0250
p=0.0034
ns.
serum level [pg/ml]
200
150
100
50
0
baseline
3 months
6 months
pre G-CSF
post G-CSF

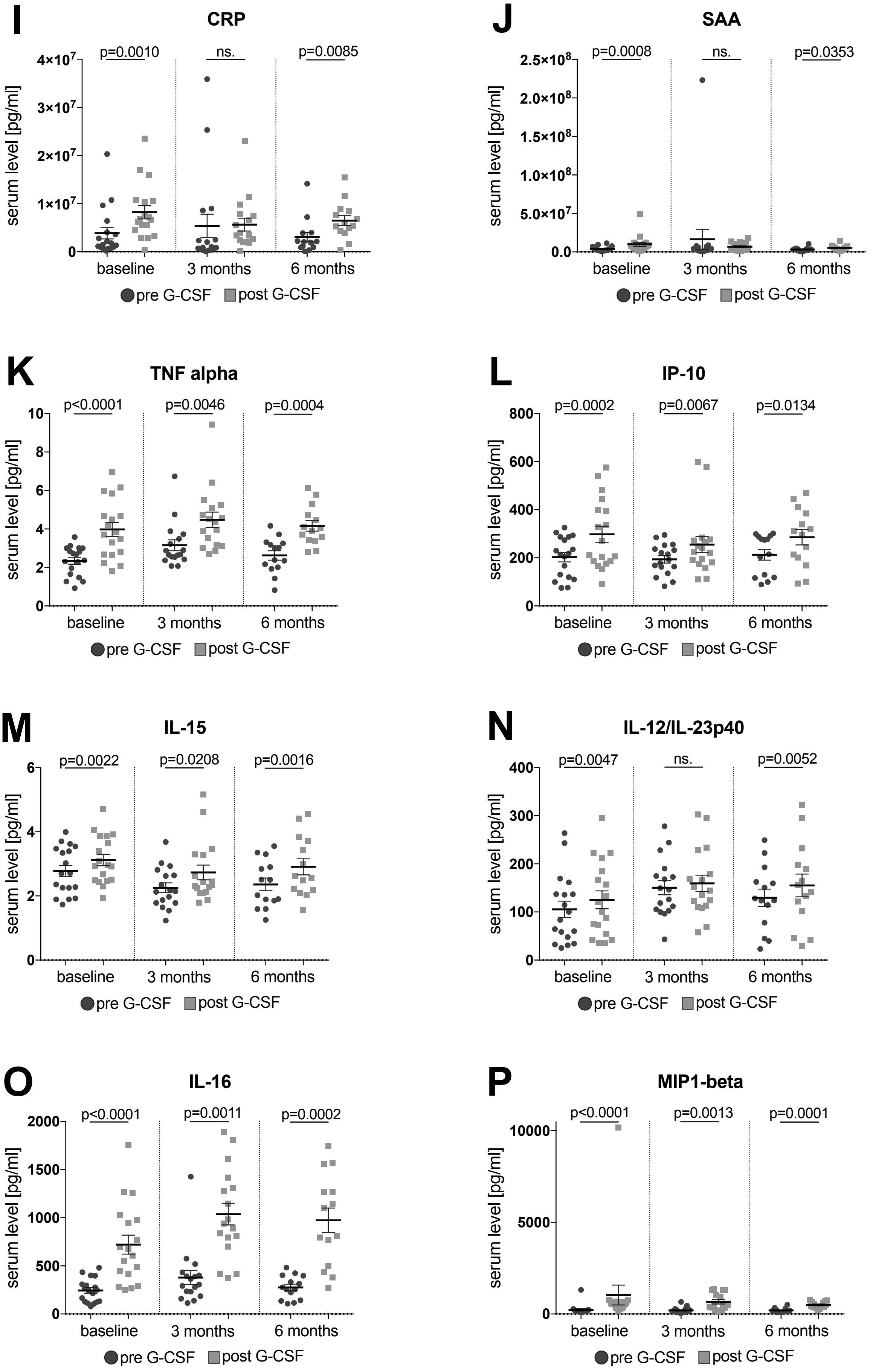
I
CRP
p=0.0010
ns.
p=0.0085
4×10^7
3×10^7
2×10^7
1×10^7
0
serum level [pg/ml]
baseline
3 months
6 months
pre G-CSF
post G-CSF
J
SAA
p=0.0008
ns.
p=0.0353
2.5×10^8
2.0×10^8
1.5×10^8
1.0×10^8
5.0×10^7
0.0
serum level [pg/ml]
baseline
3 months
6 months
pre G-CSF
post G-CSF
K
TNF alpha
p<0.0001
p=0.0046
p=0.0004
10
8
6
4
2
0
serum level [pg/ml]
baseline
3 months
6 months
pre G-CSF
post G-CSF
L
IP-10
p=0.0002
p=0.0067
p=0.0134
800
600
400
200
0
serum level [pg/ml]
baseline
3 months
6 months
pre G-CSF
post G-CSF
M
IL-15
p=0.0022
p=0.0208
p=0.0016
6
4
2
0
serum level [pg/ml]
baseline
3 months
6 months
pre G-CSF
post G-CSF
N
IL-12/IL-23p40
p=0.0047
ns.
p=0.0052
400
300
200
100
0
serum level [pg/ml]
baseline
3 months
6 months
pre G-CSF
post G-CSF
O
IL-16
p<0.0001
p=0.0011
p=0.0002
2000
1500
1000
500
0
serum level [pg/ml]
baseline
3 months
6 months
pre G-CSF
post G-CSF
P
MIP1-beta
p<0.0001
p=0.0013
p=0.0001
10000
5000
0
serum level [pg/ml]
baseline
3 months
6 months
pre G-CSF
post G-CSF

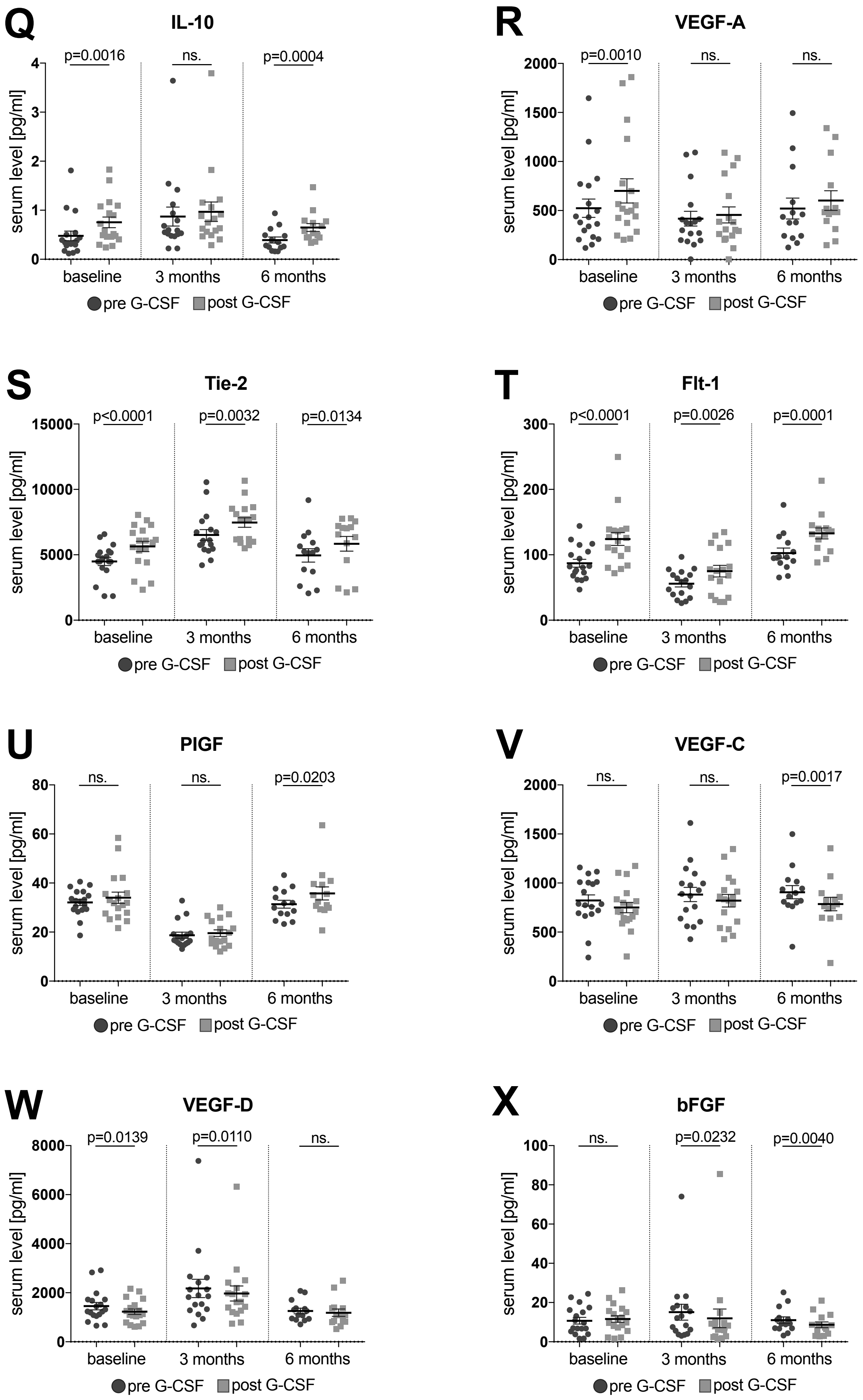
Q
IL-10
p=0.0016
ns.
p=0.0004
serum level [pg/ml]
baseline
3 months
6 months
pre G-CSF
post G-CSF
R
VEGF-A
p=0.0010
ns.
ns.
serum level [pg/ml]
baseline
3 months
6 months
pre G-CSF
post G-CSF
S
Tie-2
p<0.0001
p=0.0032
p=0.0134
serum level [pg/ml]
baseline
3 months
6 months
pre G-CSF
post G-CSF
T
Flt-1
p<0.0001
p=0.0026
p=0.0001
serum level [pg/ml]
baseline
3 months
6 months
pre G-CSF
post G-CSF
U
PlGF
ns.
ns.
p=0.0203
serum level [pg/ml]
baseline
3 months
6 months
pre G-CSF
post G-CSF
V
VEGF-C
ns.
ns.
p=0.0017
serum level [pg/ml]
baseline
3 months
6 months
pre G-CSF
post G-CSF
W
VEGF-D
p=0.0139
p=0.0110
ns.
serum level [pg/ml]
baseline
3 months
6 months
pre G-CSF
post G-CSF
X
bFGF
ns.
p=0.0232
p=0.0040
serum level [pg/ml]
baseline
3 months
6 months
pre G-CSF
post G-CSF

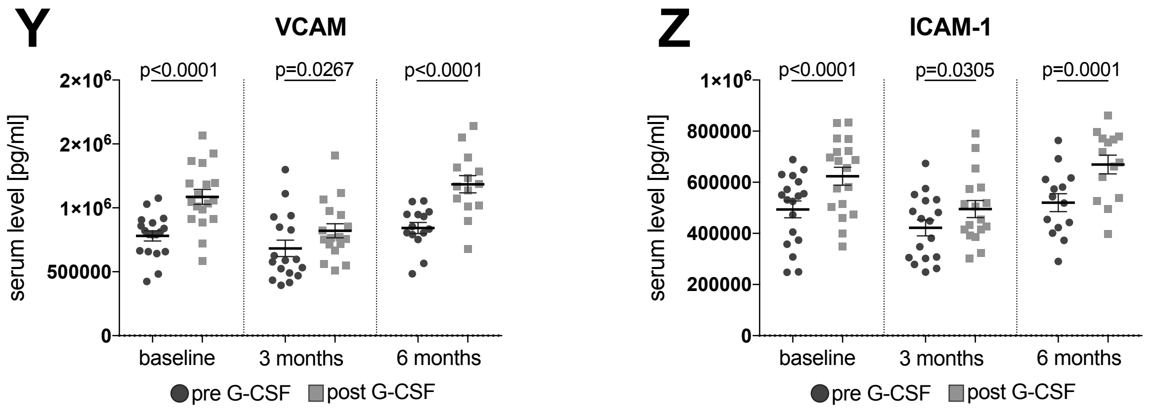


**Figure S3: Immediate effect of G-CSF on cytokine** levels (A-P pro-inflammatory and Q anti-inflammatory cytokines, R-X markers of angiogenesis, and Y-Z vascular injury) assessed by comparing levels (pg/ml) two 2 days before and 1 day after five-day G-CSF application at baseline (18 patients), after 3 months (17 patients), and after 6 months (14 patients) during ongoing treatment. Data are presented as scatter dot plot with mean + SEM. Paired Wilcoxon, p-value (two-tailed) significant at $p<0.05$. T-tests were corrected for multiple testing by FDR-adjusted p-values (q-values, given in Table 3).
